# Tetra­aqua­bis[3-(4-pyrid­yl)benzoato-κ*N*]nickel(II)

**DOI:** 10.1107/S1600536809051204

**Published:** 2009-11-28

**Authors:** Qiang-Xin Wang, Ming-Hua Zeng, Seik Weng Ng

**Affiliations:** a School of Chemistry & Chemical Engineering, Guangxi Normal University, 541004 Guilin 541004, People’s Republic of China; b Department of Chemistry, University of Malaya, 50603 Kuala Lumpur, Malaysia

## Abstract

The Ni^II^ atom in the title compound, [Ni(C_12_H_8_NO_2_)_2_(H_2_O)_4_], exists in an all-*trans* octa­hedral coordination environment. The 3-(4-pyrid­yl)benzoate ligand binds to Ni atom through the pyridyl N atom; the pyridine and benzene rings are oriented at a dihedral angle of 26.27 (10)°. Adjacent complexes are linked by O—H⋯O hydrogen bonds, forming a three-dimensional network. The metal atom lies on a special position of 2 site symmetry in the crystal structure.

## Related literature

The 3-(pyridin-4-yl)benzoate unit is fairly rigid like the nicotinate unit, which also forms a similar zwitterionic nickel derivative; see: Batten & Harris (2001 ▶).
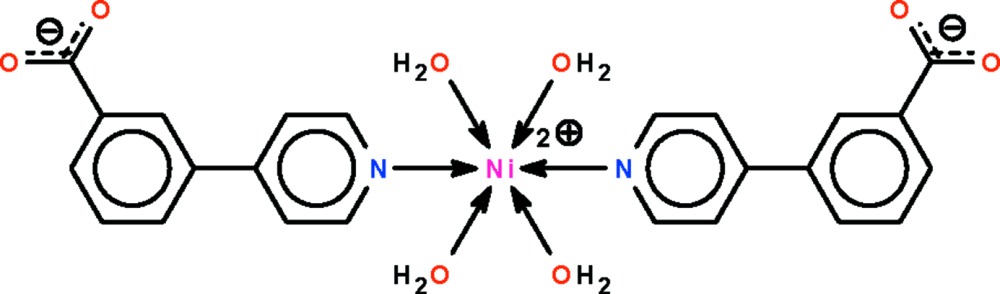


## Experimental

### 

#### Crystal data

[Ni(C_12_H_8_NO_2_)_2_(H_2_O)_4_]
*M* *_r_* = 527.16
Monoclinic, 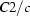

*a* = 24.564 (3) Å
*b* = 7.0520 (8) Å
*c* = 13.781 (2) Å
β = 113.325 (2)°
*V* = 2192.1 (4) Å^3^
*Z* = 4
Mo *K*α radiation
μ = 0.94 mm^−1^
*T* = 173 K
0.47 × 0.31 × 0.08 mm

#### Data collection

Bruker APEXII diffractometer
Absorption correction: multi-scan (*SADABS*; Sheldrick, 1996 ▶) *T*_min_ = 0.666, *T*_max_ = 0.929
5587 measured reflections
2360 independent reflections
1977 reflections with *I* > 2σ(*I*)
*R*_int_ = 0.023

#### Refinement

*R*[*F* ^2^ > 2σ(*F* ^2^)] = 0.031
*wR*(*F* ^2^) = 0.090
*S* = 1.09
2360 reflections
175 parameters
4 restraints
H atoms treated by a mixture of independent and constrained refinement
Δρ_max_ = 0.38 e Å^−3^
Δρ_min_ = −0.27 e Å^−3^

### 

Data collection: *APEX2* (Bruker, 2004 ▶); cell refinement: *SAINT* (Bruker, 2004 ▶); data reduction: *SAINT*; program(s) used to solve structure: *SHELXS97* (Sheldrick, 2008 ▶); program(s) used to refine structure: *SHELXL97* (Sheldrick, 2008 ▶); molecular graphics: *X-SEED* (Barbour, 2001 ▶); software used to prepare material for publication: *publCIF* (Westrip, 2009 ▶).

## Supplementary Material

Crystal structure: contains datablocks I, global. DOI: 10.1107/S1600536809051204/xu2699sup1.cif

Structure factors: contains datablocks I. DOI: 10.1107/S1600536809051204/xu2699Isup2.hkl

Additional supplementary materials:  crystallographic information; 3D view; checkCIF report

## Figures and Tables

**Table 1:** Selected bond lengths (Å)

Ni1—O1*W*	2.0627 (14)
Ni1—O2*W*	2.0811 (14)
Ni1—N1	2.0931 (16)

**Table 2:** Hydrogen-bond geometry (Å, °)

*D*—H⋯*A*	*D*—H	H⋯*A*	*D*⋯*A*	*D*—H⋯*A*
O1w—H11⋯O1^i^	0.84 (1)	1.88 (1)	2.682 (2)	160 (3)
O1w—H12⋯O2^ii^	0.83 (1)	1.91 (1)	2.734 (2)	170 (2)
O2w—H21⋯O1^iii^	0.84 (1)	1.93 (1)	2.732 (2)	159 (2)
O2w—H22⋯O2^iv^	0.83 (1)	1.88 (1)	2.711 (2)	177 (3)

Symmetry codes: (i) 
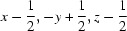
; (ii) 
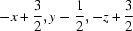
; (iii) 
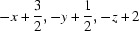
; (iv) 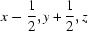.

**Fig. 1.**
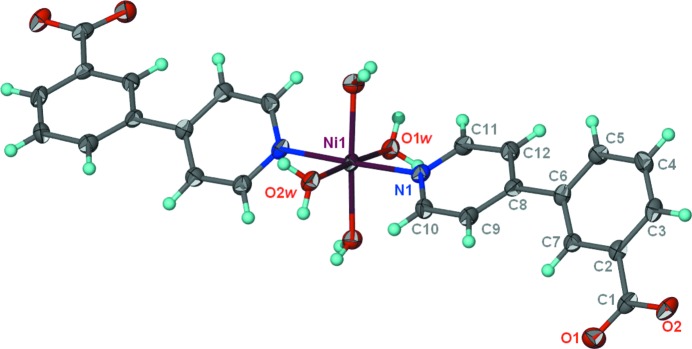
Thermal ellipsoid plot (Barbour, 2001) of Ni(H2O)4(C12H2NO2)2 at the 70% probability level; hydrogen atoms are drawn as sphere of arbitrary radius.

## supplementary crystallographic information

### Experimental

3-(Pyridin-4-yl)benzoic acid was purchased from a chemical supplier. The reagent (0.199 g, 1 mmol) and sodium hydroxide (0.040 g, 1 mmol) were mixed with nickel(II) nitrate hexahydrate (0.150 g, 0.5 mmol) in water (10 ml). The mixture was placed in a 15 ml Teflon-lined autoclave and heated at 423 K for 48 h. The autoclave was cooled over 12 h at a rate of 5 K an hour. Green crystals were isolated by hand (yield *ca* 60% based on Ni).

### Refinement

Carbon-bound hydrogen atoms were generated geometrically and were constrained to ride on their parent atoms [C–H = 0.95 Å; *U*_iso_(H) =1.2*U*_eq_(C)]. The water H-atoms were located in a difference Fourier map, and were refined with a distance restraint of O–H 0.84±0.01 Å; their temperature factors were refined.

### Crystal data

**Table:**

[Ni(C_12_H_8_NO_2_)_2_(H_2_O)_4_]	*F*(000) = 1096
*M**_r_* = 527.16	*D*_x_ = 1.597 Mg m^−^^3^
Monoclinic, *C*2/*c*	Mo *K*α radiation, λ = 0.71073 Å
Hall symbol: -C 2yc	Cell parameters from 996 reflections
*a* = 24.564 (3) Å	θ = 3.0–26.9°
*b* = 7.0520 (8) Å	µ = 0.94 mm^−^^1^
*c* = 13.781 (2) Å	*T* = 173 K
β = 113.325 (2)°	Prism, green
*V* = 2192.1 (4) Å^3^	0.47 × 0.31 × 0.08 mm
*Z* = 4	

### Data collection

**Table:**

Bruker APEXII diffractometer	2360 independent reflections
Radiation source: fine-focus sealed tube	1977 reflections with *I* > 2σ(*I*)
graphite	*R*_int_ = 0.023
φ and ω scans	θ_max_ = 27.0°, θ_min_ = 1.8°
Absorption correction: multi-scan (*SADABS*; Sheldrick, 1996)	*h* = −10→31
*T*_min_ = 0.666, *T*_max_ = 0.929	*k* = −8→8
5587 measured reflections	*l* = −17→10

### Refinement

**Table:**

Refinement on *F*^2^	Primary atom site location: structure-invariant direct methods
Least-squares matrix: full	Secondary atom site location: difference Fourier map
*R*[*F*^2^ > 2σ(*F*^2^)] = 0.031	Hydrogen site location: inferred from neighbouring sites
*wR*(*F*^2^) = 0.090	H atoms treated by a mixture of independent and constrained refinement
*S* = 1.09	*w* = 1/[σ^2^(*F*_o_^2^) + (0.0539*P*)^2^ + 0.5684*P*] where *P* = (*F*_o_^2^ + 2*F*_c_^2^)/3
2360 reflections	(Δ/σ)_max_ = 0.001
175 parameters	Δρ_max_ = 0.38 e Å^−^^3^
4 restraints	Δρ_min_ = −0.27 e Å^−^^3^

### Fractional atomic coordinates and isotropic or equivalent isotropic displacement parameters (Å^2^)

**Table:**

	*x*	*y*	*z*	*U*_iso_*/*U*_eq_	
Ni1	0.5000	0.30069 (5)	0.7500	0.01561 (13)	
N1	0.58142 (7)	0.3092 (2)	0.73473 (13)	0.0179 (3)	
O1	0.90563 (6)	0.2232 (2)	0.96391 (12)	0.0266 (3)	
O2	0.96160 (6)	0.29413 (19)	0.87580 (12)	0.0242 (3)	
O1W	0.46891 (6)	0.0848 (2)	0.64084 (11)	0.0199 (3)	
O2W	0.53247 (6)	0.5047 (2)	0.86819 (11)	0.0208 (3)	
C1	0.91223 (9)	0.2740 (3)	0.88189 (16)	0.0197 (4)	
C2	0.85662 (8)	0.3156 (3)	0.78537 (16)	0.0172 (4)	
C3	0.85969 (8)	0.3652 (3)	0.69003 (17)	0.0208 (4)	
H3	0.8970	0.3732	0.6845	0.025*	
C4	0.80768 (8)	0.4029 (3)	0.60302 (16)	0.0220 (4)	
H4	0.8096	0.4366	0.5377	0.026*	
C5	0.75295 (8)	0.3919 (3)	0.61016 (15)	0.0205 (4)	
H5	0.7178	0.4188	0.5500	0.025*	
C6	0.74932 (8)	0.3414 (3)	0.70535 (15)	0.0176 (4)	
C7	0.80178 (8)	0.3043 (3)	0.79252 (16)	0.0173 (4)	
H7	0.8000	0.2706	0.8580	0.021*	
C8	0.69121 (8)	0.3288 (3)	0.71474 (16)	0.0173 (4)	
C9	0.68658 (8)	0.3575 (3)	0.81138 (16)	0.0198 (4)	
H9	0.7210	0.3856	0.8727	0.024*	
C10	0.63215 (9)	0.3452 (3)	0.81801 (16)	0.0205 (4)	
H10	0.6305	0.3632	0.8851	0.025*	
C11	0.58576 (9)	0.2824 (3)	0.64134 (16)	0.0199 (4)	
H11A	0.5505	0.2566	0.5811	0.024*	
C12	0.63867 (8)	0.2903 (3)	0.62852 (16)	0.0198 (4)	
H12A	0.6392	0.2694	0.5608	0.024*	
H11	0.4524 (11)	0.124 (4)	0.5786 (11)	0.048 (9)*	
H12	0.4935 (8)	0.004 (3)	0.6396 (17)	0.023 (6)*	
H21	0.5473 (10)	0.452 (3)	0.9275 (11)	0.030 (7)*	
H22	0.5096 (10)	0.591 (3)	0.869 (2)	0.043 (8)*	

### Atomic displacement parameters (Å^2^)

**Table:**

	*U*^11^	*U*^22^	*U*^33^	*U*^12^	*U*^13^	*U*^23^
Ni1	0.01032 (18)	0.0186 (2)	0.0183 (2)	0.000	0.00608 (14)	0.000
N1	0.0125 (7)	0.0184 (8)	0.0222 (9)	−0.0001 (6)	0.0061 (7)	0.0022 (7)
O1	0.0189 (7)	0.0361 (9)	0.0215 (8)	−0.0012 (6)	0.0043 (6)	0.0023 (6)
O2	0.0118 (7)	0.0227 (8)	0.0362 (9)	0.0008 (5)	0.0077 (6)	0.0012 (6)
O1W	0.0155 (7)	0.0222 (8)	0.0216 (8)	0.0010 (6)	0.0071 (6)	−0.0019 (6)
O2W	0.0156 (7)	0.0225 (8)	0.0226 (8)	0.0028 (6)	0.0056 (6)	−0.0022 (6)
C1	0.0154 (9)	0.0154 (9)	0.0259 (11)	−0.0002 (7)	0.0056 (8)	−0.0042 (8)
C2	0.0149 (9)	0.0145 (9)	0.0214 (10)	−0.0026 (7)	0.0064 (8)	−0.0045 (8)
C3	0.0149 (9)	0.0215 (10)	0.0294 (11)	−0.0027 (8)	0.0122 (8)	−0.0026 (8)
C4	0.0212 (10)	0.0277 (11)	0.0202 (10)	−0.0032 (8)	0.0115 (8)	−0.0003 (9)
C5	0.0158 (9)	0.0234 (10)	0.0206 (10)	−0.0007 (8)	0.0054 (8)	−0.0002 (8)
C6	0.0135 (9)	0.0176 (9)	0.0223 (10)	−0.0020 (7)	0.0078 (8)	−0.0033 (7)
C7	0.0156 (9)	0.0179 (9)	0.0187 (9)	0.0002 (7)	0.0071 (8)	0.0002 (8)
C8	0.0149 (9)	0.0163 (9)	0.0205 (10)	0.0014 (7)	0.0069 (8)	0.0024 (7)
C9	0.0130 (9)	0.0242 (10)	0.0204 (10)	0.0011 (8)	0.0047 (8)	0.0004 (8)
C10	0.0175 (9)	0.0255 (10)	0.0198 (10)	0.0014 (8)	0.0090 (8)	0.0003 (8)
C11	0.0139 (9)	0.0237 (10)	0.0210 (10)	−0.0004 (8)	0.0057 (8)	0.0008 (8)
C12	0.0167 (10)	0.0230 (10)	0.0196 (10)	−0.0007 (8)	0.0071 (8)	−0.0007 (8)

### Geometric parameters (Å, °)

**Table:**

Ni1—O1W	2.0627 (14)	C3—C4	1.388 (3)
Ni1—O1W^i^	2.0627 (14)	C3—H3	0.9500
Ni1—O2W	2.0811 (14)	C4—C5	1.389 (3)
Ni1—O2W^i^	2.0811 (14)	C4—H4	0.9500
Ni1—N1^i^	2.0931 (16)	C5—C6	1.396 (3)
Ni1—N1	2.0931 (16)	C5—H5	0.9500
N1—C10	1.342 (3)	C6—C7	1.395 (3)
N1—C11	1.346 (2)	C6—C8	1.486 (3)
O1—C1	1.256 (3)	C7—H7	0.9500
O2—C1	1.256 (2)	C8—C12	1.392 (3)
O1W—H11	0.838 (10)	C8—C9	1.396 (3)
O1W—H12	0.834 (10)	C9—C10	1.378 (3)
O2W—H21	0.840 (10)	C9—H9	0.9500
O2W—H22	0.833 (10)	C10—H10	0.9500
C1—C2	1.511 (3)	C11—C12	1.380 (3)
C2—C3	1.390 (3)	C11—H11A	0.9500
C2—C7	1.391 (3)	C12—H12A	0.9500
			
O1W—Ni1—O1W^i^	84.85 (8)	C4—C3—C2	119.26 (17)
O1W—Ni1—O2W	176.09 (6)	C4—C3—H3	120.4
O1W^i^—Ni1—O2W	91.32 (6)	C2—C3—H3	120.4
O1W—Ni1—O2W^i^	91.32 (6)	C3—C4—C5	120.92 (18)
O1W^i^—Ni1—O2W^i^	176.09 (6)	C3—C4—H4	119.5
O2W—Ni1—O2W^i^	92.51 (8)	C5—C4—H4	119.5
O1W—Ni1—N1^i^	90.11 (6)	C4—C5—C6	120.31 (18)
O1W^i^—Ni1—N1^i^	92.32 (6)	C4—C5—H5	119.8
O2W—Ni1—N1^i^	89.24 (6)	C6—C5—H5	119.8
O2W^i^—Ni1—N1^i^	88.48 (6)	C7—C6—C5	118.45 (17)
O1W—Ni1—N1	92.32 (6)	C7—C6—C8	120.35 (17)
O1W^i^—Ni1—N1	90.11 (6)	C5—C6—C8	121.19 (17)
O2W—Ni1—N1	88.48 (6)	C2—C7—C6	121.21 (18)
O2W^i^—Ni1—N1	89.24 (6)	C2—C7—H7	119.4
N1^i^—Ni1—N1	176.71 (9)	C6—C7—H7	119.4
C10—N1—C11	116.48 (16)	C12—C8—C9	116.40 (17)
C10—N1—Ni1	121.33 (13)	C12—C8—C6	122.37 (18)
C11—N1—Ni1	122.18 (13)	C9—C8—C6	121.23 (17)
Ni1—O1W—H11	113 (2)	C10—C9—C8	120.12 (18)
Ni1—O1W—H12	117.0 (16)	C10—C9—H9	119.9
H11—O1W—H12	105 (2)	C8—C9—H9	119.9
Ni1—O2W—H21	109.7 (18)	N1—C10—C9	123.49 (18)
Ni1—O2W—H22	117.8 (19)	N1—C10—H10	118.3
H21—O2W—H22	110 (2)	C9—C10—H10	118.3
O1—C1—O2	124.36 (19)	N1—C11—C12	123.49 (19)
O1—C1—C2	116.99 (17)	N1—C11—H11A	118.3
O2—C1—C2	118.65 (18)	C12—C11—H11A	118.3
C3—C2—C7	119.85 (18)	C11—C12—C8	120.03 (19)
C3—C2—C1	120.82 (17)	C11—C12—H12A	120.0
C7—C2—C1	119.32 (18)	C8—C12—H12A	120.0
			
O1W—Ni1—N1—C10	−144.22 (15)	C3—C2—C7—C6	0.1 (3)
O1W^i^—Ni1—N1—C10	−59.36 (15)	C1—C2—C7—C6	−179.93 (17)
O2W—Ni1—N1—C10	31.96 (15)	C5—C6—C7—C2	−0.4 (3)
O2W^i^—Ni1—N1—C10	124.49 (15)	C8—C6—C7—C2	−179.88 (17)
O1W—Ni1—N1—C11	36.93 (15)	C7—C6—C8—C12	−154.18 (19)
O1W^i^—Ni1—N1—C11	121.79 (15)	C5—C6—C8—C12	26.4 (3)
O2W—Ni1—N1—C11	−146.89 (15)	C7—C6—C8—C9	26.6 (3)
O2W^i^—Ni1—N1—C11	−54.36 (15)	C5—C6—C8—C9	−152.89 (19)
O1—C1—C2—C3	−177.38 (18)	C12—C8—C9—C10	0.6 (3)
O2—C1—C2—C3	3.5 (3)	C6—C8—C9—C10	179.95 (18)
O1—C1—C2—C7	2.7 (3)	C11—N1—C10—C9	0.8 (3)
O2—C1—C2—C7	−176.42 (17)	Ni1—N1—C10—C9	−178.14 (15)
C7—C2—C3—C4	0.0 (3)	C8—C9—C10—N1	−1.1 (3)
C1—C2—C3—C4	−179.89 (18)	C10—N1—C11—C12	0.0 (3)
C2—C3—C4—C5	0.1 (3)	Ni1—N1—C11—C12	178.89 (14)
C3—C4—C5—C6	−0.4 (3)	N1—C11—C12—C8	−0.4 (3)
C4—C5—C6—C7	0.5 (3)	C9—C8—C12—C11	0.1 (3)
C4—C5—C6—C8	179.99 (18)	C6—C8—C12—C11	−179.22 (18)

Symmetry codes: (i) −*x*+1, *y*, −*z*+3/2.

### Hydrogen-bond geometry (Å, °)

**Table:**

*D*—H···*A*	*D*—H	H···*A*	*D*···*A*	*D*—H···*A*
O1w—H11···O1^ii^	0.84 (1)	1.88 (1)	2.682 (2)	160 (3)
O1w—H12···O2^iii^	0.83 (1)	1.91 (1)	2.734 (2)	170 (2)
O2w—H21···O1^iv^	0.84 (1)	1.93 (1)	2.732 (2)	159 (2)
O2w—H22···O2^v^	0.83 (1)	1.88 (1)	2.711 (2)	177 (3)

Symmetry codes: (ii) *x*−1/2, −*y*+1/2, *z*−1/2; (iii) −*x*+3/2, *y*−1/2, −*z*+3/2; (iv) −*x*+3/2, −*y*+1/2, −*z*+2; (v) *x*−1/2, *y*+1/2, *z*.
